# Correlation of retinal nerve fibre layer and macular thickness with serum uric acid among type 2 diabetes mellitus

**DOI:** 10.1186/s12886-017-0486-3

**Published:** 2017-06-14

**Authors:** Munisamy-Naidu Vinuthinee-Naidu, Embong Zunaina, Anuar Azreen-Redzal, Naing Nyi-Nyi

**Affiliations:** 1 Department of Ophthalmology, School of Medical Sciences, Universiti Sains Malaysia, 16150 Kubang Kerian, Kelantan Malaysia; 2 Hospital Universiti Sains Malaysia, Jalan Raja Perempuan Zainab II, 16150 Kubang Kerian, Kelantan Malaysia; 3 Department of Ophthalmology, Hospital Sultanah Bahiyah, 05460 Alor Setar, Kedah Malaysia; 4 Unit of Biostatistics and Research Methodology, School of Medical Sciences, Universiti Sains Malaysia, 16150 Kubang Kerian, Kelantan Malaysia

**Keywords:** Diabetic retinopathy, Retinal nerve fibre layer thickness, Macular thickness, Serum uric acid, Glycosylated haemoglobin

## Abstract

**Background:**

Uric acid is a final breakdown product of purine catabolism in humans. It’s a potent antioxidant and can also act as a pro-oxidant that induces oxidative stress on the vascular endothelial cells, thus mediating progression of diabetic related diseases. Various epidemiological and experimental evidence suggest that uric acid has a role in the etiology of type 2 diabetes mellitus. We conducted a cross-sectional study to evaluate the correlation of retinal nerve fibre layer (RNFL) and macular thickness with serum uric acid in type 2 diabetic patients.

**Methods:**

A cross-sectional study was conducted in the Eye Clinic, Hospital Universiti Sains Malaysia, Kelantan between the period of August 2013 till July 2015 involving type 2 diabetes mellitus patients with no diabetic retinopathy and with non-proliferative diabetic retinopathy (NPDR). An evaluation for RNFL and macular thickness was measured using Spectralis Heidelberg optical coherence tomography. Six ml of venous blood was taken for the measurement of serum uric acid and glycosylated haemoglobin (HbA1_C_).

**Results:**

A total of 180 diabetic patients were recruited (90 patients with no diabetic retinopathy and 90 patients with NPDR) into the study. The mean level of serum uric acid for both the groups was within normal range and there was no significance difference between the two groups. Based on gender, both male and female gender showed significantly higher level of mean serum uric acid in no diabetic retinopathy group (*p* = 0.004 respectively). The mean serum uric acid was significantly higher in patient with HbA1_C_ < 6.5% (*p* < 0.031). Patients with NPDR have thicker RNFL and macular thickness compared to patients with no diabetic retinopathy. However, only the RNFL thickness of the temporal quadrant and the macular thickness of the superior outer, inferior outer and temporal outer subfields were statistically significant (*p* = 0.038, *p* = 0.004, 0.033 and <0.001 respectively). There was poor correlation between RNFL and macular thickness with serum uric acid in both the groups.

**Conclusion:**

Serum uric acid showed a poor correlation with RNFL and macular thickness among type 2 diabetic patients.

## Background

Diabetes mellitus is regarded as a pandemic, representing one of the most challenging and major public health problems of the twenty-first century. It has become a global alarming disease not sparing any country thus posing a serious threat to its economy [1]. It has been shown that, prevalence of diabetes is rapidly rising and is a major cause of morbidity and mortality [2].

Diabetic retinopathy is the commonest microvascular complication of diabetes mellitus. It remains the leading cause of preventable blindness across all age-groups and places a significant burden on health services [3]. It’s termed as a neurovascular disease which affects both the neuroretinal and microvascular component, and the former is said to be compromised early during diabetic retinopathy followed by microvascular changes [4]. The hallmark of retinal neurodegeneration is neural apoptosis of the retinal ganglion cells which are in the inner retina layer and reactive gliosis involving the astrocytes and Muller cells [5]. It is suggested that the functional changes noticed before the vascular pathology develops are due to direct effect of diabetes on the neural retina instead of breakdown of the blood retinal barrier [4].

Several studies have used electroretinogram (ERG) on the retinal nerve fibre layer (RNFL). An abnormal result suggests early retinal neural dysfunction which in later stages progress to neurodegeneration [6, 7]. It’s also accompanied by deficits in contrast sensitivity, loss of dark adaptation and colour vision disturbances [8]. Risk factors for RNFL degeneration in a diabetic patient are hyperglycemia, oxidative stress and advanced glycation end products [9]. Macular thickness in the presence of diabetic retinopathy has been shown to be thicker compared to normal population. This is also seen in diabetic patient with no demonstrable evidence of retinopathy or macular oedema [10].

**Fig. 1:**
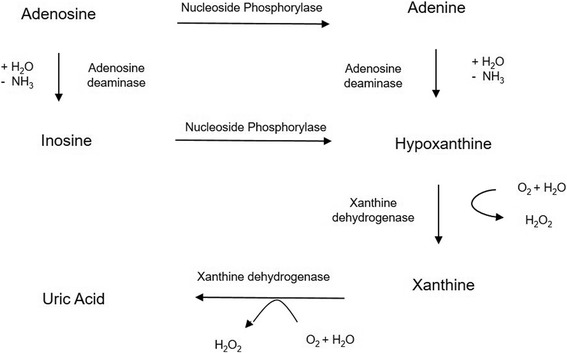
Purine metabolic pathway. Adapted from Jianfei Xia et al. [12]

Serum uric acid is the product of endogenous and exogenous purine metabolism and its derivatives [11]. In the purine metabolic pathway (Fig. 1), adenosine has vasoactive properties that play a role in retinal blood flow. It generates superoxide nitric oxide which affect the retinal circulation by causing capillary occlusion, apoptosis of pericytes and basement membrane thickening [12]. Xanthine, a substrate of xanthine oxidase, enhances superoxide generation, causing microvascular dysfunction and exert tissue damage resulting in lipid and protein peroxidation [12]. These changes are seen in pathogenesis of diabetic retinopathy. Various epidemiological and experimental evidence suggest that uric acid has a role in the aetiology of type 2 diabetes mellitus [11–13].

From as early as 1950, Griffiths M [14] reported the diabetogenic action of serum uric acid and suggested that it’s levels are associated with an increased risk of type 2 diabetic complications. Several other studies have also observed similar findings [15, 16]. Goldberg RB [17], attributed proatherogenic properties of serum uric acid to be responsible for the pathogenesis of diabetic retinopathy and other diabetic vascular complications. These include activation of endothelial cells & platelets and increased platelet adhesiveness. Navin S et al. [11] also concluded that poor glycemic control in type 2 diabetes mellitus is associated with an increased serum uric acid level and dyslipidemia, which could be the initial ongoing biochemical change in the complication of diabetes.

In view of numerous evidence suggesting the role of uric acid in diabetic patients, our study was conducted to correlate the RNFL thickness and macular thickness with serum uric acid among type 2 diabetes mellitus patients. To the best of our knowledge, there are no studies done that correlate serum uric acid with RNFL and macular thickness in diabetic patients.

## Methods

A cross-sectional study was carried out on 180 patients with type 2 diabetic mellitus [90 patients with no diabetic retinopathy and 90 patients with non-proliferative diabetic retinopathy (NPDR)] who attended the Eye Clinic, Hospital Universiti Sains Malaysia, Kelantan, Malaysia from August 2013 to July 2015.

Type 2 diabetes mellitus patients aged between 40 and 65 years old with clear media were included. Those patients with optic nerve, retina and macular pathology, NPDR with moderate and severe diabetic macular oedema, previous history of laser, history of intraocular surgery, renal disease and hyperuricemia were excluded from this study.

The demographic data (age, gender and ethnicity) was obtained either from the patient or their medical record. A thorough slit lamp and fundus examination was performed by one identified ophthalmologist to confirm the diagnosis. The classification of diabetic retinopathy was based on the International Diabetic Retinopathy Severity scales [18]. Only one eye of the worst severity was selected.

**Fig. 2:**
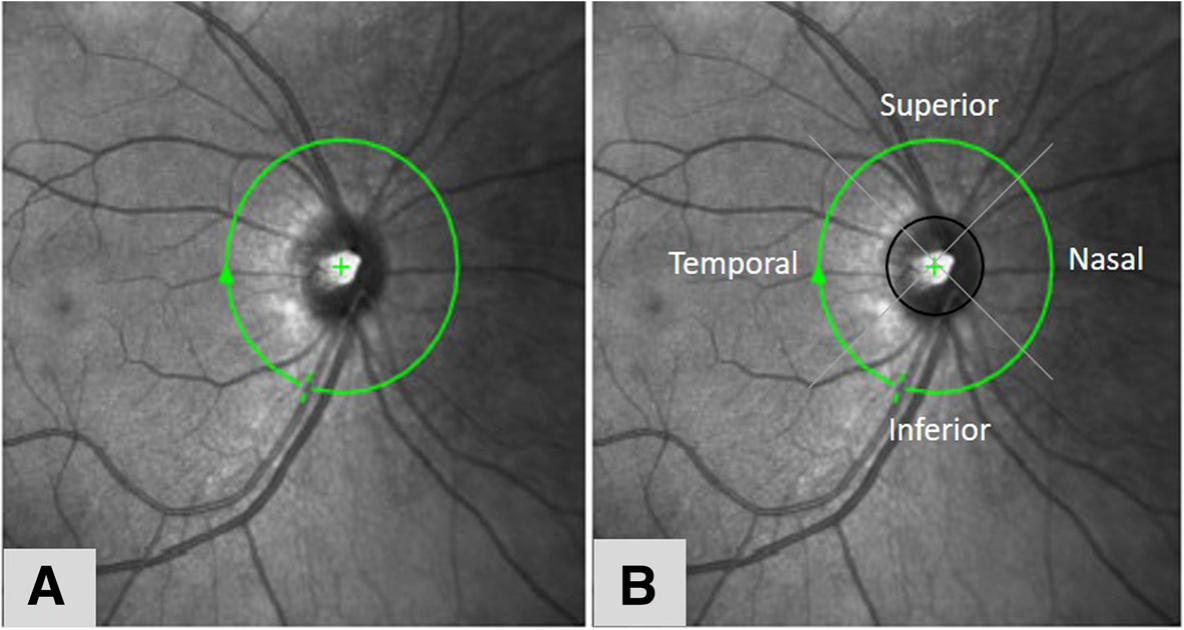
Centration of peripapillary ring (**a**) and quadrants (**b**) for retinal nerve fibre layer measurement

**Fig. 3:**
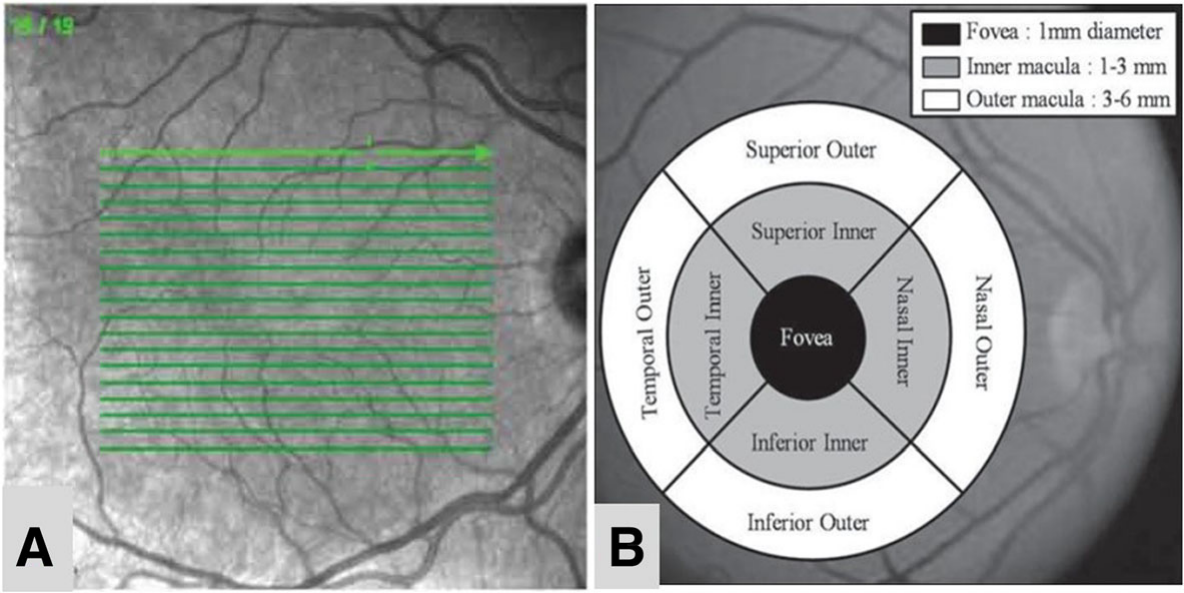
The ‘6 mm fast macular mapping’ with 19 raster line centered at the fovea (**a**) and the Early Treatment Diabetic Retinopathy Study (ETDRS) grid dividing the macula into 9 subfields (**b**) for macular thickness measurement

The RNFL and macular thickness was measured using the Spectralis Spectral-Domain Optical Coherence Tomography (SD-OCT) (Heidelberg Engineering, Heidelberg, Germany). For RNFL thickness analysis, a 3.45 mm diameter peripapillary ring was measured (Fig. 2a). All the four quadrants were taken for analysis in this study; superior, inferior, temporal and nasal (Fig. 2b). The macular thickness was determined by using the ‘6 mm fast macular mapping’ scanning pattern. It consists of a high resolution 19 raster line scan protocol that was applied on an area centered on the fovea with the horizontal lines spaced 240 μm apart (Fig. 3a). The Early Treatment Diabetic Retinopathy Study (ETDRS) grid was applied dividing the macula into 9 subfields, encircled by rings of 1, 3 and 6 mm in diameter. All the subfields were taken for analysis in this study: fovea; superior inner, inferior inner, temporal inner, nasal inner in the second ring; superior outer, inferior outer, temporal outer and nasal outer in the outer ring (Fig. 3b).

Six ml of venous blood was drawn from the median cubital vein for measurement of serum uric acid and glycosylated haemoglobin (HbA1_C_). The reference normal range for serum uric acid measured in Hospital Universiti Sains Malaysia differs in each gender: Male 180–420 μmol/L; Female 150–360 μmol/L.

### Statistical methods

The statistical analysis was carried out using Statistical Package for Social Sciences (SPSS) Version 22. All values were tested for normal distribution and equal variances. Chi Square test was used for comparison of gender and ethnicity. Independent t-test was used for comparison of age, HbA1_C_, serum uric acid, RNFL thickness and macular thickness. Significance of difference in values was determined by the ‘p’ value <0.05. The correlation between RNFL thickness and macular thickness with serum uric acid was tested using Pearson correlation. Significance of correlation was decided based on ‘r’ [19] and ‘p’ values.

## Results

The distribution of demographic data is shown in Table 1. The male and female gender were not equally distributed among both groups; however, it was not significant. The majority of the patients were Malay in both the groups. Both the groups showed a poor diabetic control with mean HbA1_C_ more than 6.5%. The HbA1_C_ was significantly higher in NPDR (9.59%, standard deviation (SD) 2.27) compared to no diabetic retinopathy (7.84%, SD 2.01) (*p* < 0.001).

**Table 1:** Distribution of age, gender, ethnic group and mean HbA1_C_

Characteristics	No DR *n* = 90	NPDR *n* = 90	t statistic (df)	*p* value
Age (years)				
Mean (SD)	52.16 (5.87)	51.21 (5.20)	1.142	0.225 ^a^
Gender (n, %)				
Male	51 (56.70)	40 (44.40)	2.689	0.101^b^
Female	39 (43.30)	50 (55.60)		
Ethnicity (n, %)				
Malay	73 (81.10)	75 (83.30)	3.958	0.138^b^
Chinese	11 (12.20)	14 (15.60)		
Indian	6 (6.70)	1 (1.10)		
HbA1_C_ (%)				
Mean (SD)	7.84 (2.01)	9.59 (2.27)	5.467	< 0.001^a^

*DR* Diabetic Retinopathy, *NPDR* Non-Proliferative Diabetic Retinopathy, *SD* Standard Deviation

^a^Independent T-test, ^b^Chi Square, *p* value <0.05 significant

The mean level of serum uric acid for both the groups was within normal range but at the upper limit. There was no statistically significant difference between the groups (*p* = 0.220) (Table 2). However, based on gender, both male and female patients with no diabetic retinopathy showed a significantly higher level of mean serum uric acid compared to those in NPDR group (*p* = 0.004 respectively). The mean level of serum uric acid was significantly higher in those patients with HbA1c < 6.5% compared to those with HbA1c **≥** 6.5% (*p* = 0.031) (Table 3).

**Table 2:** Serum uric acid in no diabetic retinopathy and NPDR

Serum uric acid (μmol/L)	Mean (SD)		Mean difference (95% CI)	t statistics (df)	*p* value
	No DR	NPDR			
Total	377.53 (95.41)	360.09 (94.69)	(−10.517, 45.406)	1.231	0.220
Male	402.55 (102.44)	391.75 (88.15)	(−95.068, 18.704)	−2.958	0.004
Female	344.82 (74.63)	334.76 (92.89)	(−96.402, −19.055)	−2.967	0.004

Independent t-test, *p*-value <0.05 significant

*DR* Diabetic Retinopathy, *NPDR* Non-Proliferative Diabetic Retinopathy, *SD* Standard Deviation, *CI* Confidence Interval

**Table 3:** Comparison of mean serum uric acid with HbA1_C_

	Mean (SD)		Mean difference (95% CI)	t statistic (df)	*p* value
	HbA1_C_				
	< 6.5%	≥ 6.5%			
Serum uric acid (μmol/L)	403.70 (97.91)	362.11 (93.50)	41.577 (3.885, 79.269)	2.177	0.031

Independent T-test, *p*-value <0.05 significant

*SD* standard deviation, *CI* confidence interval

Patients with NPDR generally have thicker RNFL and macular thickness compared to patients with no diabetic retinopathy. However, only the RNFL thickness of the temporal quadrant and the macular thickness of the superior outer, inferior outer and temporal outer subfields were statistically significant (*p* = 0.038, *p* = 0.004, 0.033 and <0.001 respectively) (Table 4).

**Table 4:** RNFL and macular thickness in no diabetic retinopathy and NPDR

Quadrants / Subfields	Mean (SD)		Mean difference (95% CI)	t statistics (df)	*p* value
	No DR	NPDR			
RNFL thickness (μm)					
Superior	107.76 (21.92)	111.52 (22.31)	−9.947, 3.597	−1.126	0.262
Inferior	108.16 (22.92)	108.81 (25.01)	−7.700, 6.968	−0.183	0.855
Temporal	71.10 (13.71)	75.02 (11.33)	−7.621, −0.226	−2.092	0.038
Nasal	73.72 (15.13)	73.79 (15.19)	−4.015, 5.082	0.231	0.817
Macular thickness (μm)					
Fovea	262.57 (23.44)	262.86 (29.34)	−8.110, 7.522	−0.073	0.942
Superior inner	330.63 (17.25)	333.64 (28.34)	−9.911, 3.889	−0.861	0.391
Superior outer	288.37 (16.05)	298.07 (27.20)	−16.268, −3.313	−2.291	0.004
Inferior inner	327.62 (19.33)	330.41 (28.22)	−9.904, 4.326	−0.773	0.440
Inferior outer	278.93 (22.71)	286.98 (27.20)	−15.415, −0.674	−2.154	0.033
Temporal inner	320.07 (18.20)	323.68 (27.93)	−10.547, 3.323	−1.028	0.306
Temporal outer	274.11 (14.05)	284.90 (24.38)	−16.641, −4.936	−3.638	<0.001
Nasal inner	334.70 (20.00)	366.69 (26.62)	−100.056, 36.079	−0.927	0.355
Nasal outer	306.32 (17.90)	311.59 (24.49)	−11.576, 1.043	−0.165	0.101

Independent t-test, *p*-value <0.05 significant

*DR* Diabetic Retinopathy, *NPDR* Non-Proliferative Diabetic Retinopathy, *RNFL* Retinal Nerve Fibre Layer, *SD* Standard Deviation, *CI* Confidence Interval

The RNFL thickness showed a poor negative correlation with serum uric acid in all the quadrants for both the groups except the RNFL thickness of the temporal quadrant of no diabetic retinopathy group showed a poor positive correlation (Table 5). All the macular subfields in the no diabetic retinopathy group showed a poor negative correlation with serum uric acid except the fovea subfield showed poor positive correlation. Whereas, all the macular subfields in the NPDR group showed a poor positive correlation except superior outer subfield showed a poor negative correlation (Table 5).

**Table 5:** Correlation of RNFL and macular thickness with serum uric acid among type 2 diabetes mellitus patients

Quadrants / Subfields	Serum uric acid (μmol/L)			
	No DR		NPDR	
	r	*p*-value	r	*p*-value
RNFL thickness (μm)				
Superior	−0.078	0.467	−0.113	0.288
Inferior	−0.146	0.169	−0.086	0.419
Temporal	0.064	0.548	−0.067	0.532
Nasal	−0.034	0.751	−0.069	0.519
Macular thickness (μm)				
Fovea	0.012	0.912	0.219	0.038
Superior inner	−0.095	0.374	0.034	0.749
Superior outer	−0.110	0.304	−0.019	0.857
Inferior inner	−0.063	0.552	0.264	0.012
Inferior outer	−0.225	0.033	0.016	0.885
Temporal inner	0.000	0.999	0.176	0.097
Temporal outer	−0.113	0.287	0.073	0.493
Nasal inner	−0.013	0.903	0.066	0.538
Nasal outer	−0.085	0.426	0.010	0.929

Pearson correlation; r: correlation coefficient, *p*-value <0.05 significant

*DR* Diabetic Retinopathy, *NPDR* Non-Proliferative Diabetic Retinopathy, *RNFL* Retinal Nerve Fibre Layer

## Discussion

Uric acid is a final breakdown product of purine catabolism in humans. It’s a potent anti-oxidant and can also act as a pro-oxidant that induces oxidative stress on the vascular endothelial cells, thus mediating progression of diabetic related diseases [20]. Various epidemiological and experimental evidence suggest that uric acid has a role in the etiology of type 2 diabetes mellitus [11–13]. We conducted a cross-sectional study to evaluate the correlation of RNFL and macular thickness with serum uric acid in type 2 diabetic patients.

The mean age of our study participants ranged between 51 to 52 years old. This was consistent with several other studies [21, 22]. In contrast, Eydis Olafsdottir et al. [23] showed the prevalence of no diabetic retinopathy and those with retinopathy in diabetic patients was slightly in the older age group. However, Hansson-Lundblad et al. [24], reported there is no association between age and retinopathy. Perhaps, duration of diabetes, is an independent risk factor for occurrence of retinopathy [21, 23]. Both the gender was not equally distributed in our study. This was like previous studies that reported no significant differences between retinopathy and gender [21, 25].

Most of the participants in our study were Malay. Malaysia has a multiethnic population with three main races; Malay, Chinese and Indian. However, Kelantan is a predominantly Malay village in the north-eastern state of the Peninsular. This perhaps explains the preponderance of Malay participants in our study. From our findings, the NPDR group had poor glycemic control. This was like other studies done at various places [26, 27].

There are several studies that have linked serum uric acid with the pathogenesis and progression of diabetic retinopathy [13, 28]. In our study, total serum uric acid was higher in no diabetic retinopathy group, but not statistically significant. The serum uric acid was significantly higher in patients with HbA1_C_ < 6.5%. This in accordance with previous studies that stated elevated serum uric acid levels during the early stage of impaired glucose metabolism is said to predict the onset of the type 2 diabetes and has been linked to both micro and macrovascular complications [29, 30]. Bonakdaran S et al. [31], noted a significant correlation between hyperuricemia and HbA1_C_. Dehghan A et al. [16] reported high serum uric acid level precedes hyperinsulinemia and diabetes inducing endothelial dysfunction and oxidative stress.

Few studies involving type 2 diabetic patients showed serum uric acid levels correspond to the severity of diabetic retinopathy [32–34]. The serum uric acid level was showed to increase gradually with increase of severity of diabetic retinopathy. Causevic A et al. [35] reported that the serum uric acid level was increased in type 2 diabetes mellitus and associated with insulin resistant syndrome. Moreover, Ashakiran S. et al. [36], noticed that the compensatory hyperinsulinemia [insulin resistant] showed an antiuricosuric effect on the kidneys which lead to increase serum uric acid level.

In contrast, Nan H et al. [37] mentioned that the serum uric acid and fasting plasma glucose level increases in non-diabetic individuals but showed lower level in diabetic patient. However, Olukoga AO et al. [38] and Segato T et al. [39], reported no significant association between serum uric acid and diabetic retinopathy. Pfister R et al. [40], tested eight common genetic variants which were identified as determinant of serum uric acid level on diabetic patients involving a large cohort of European descent. Their results do not support the association of serum uric acid in the development of type 2 diabetes mellitus.

The male gender had higher level of serum uric acid compared to the female gender. We also observed, the mean serum uric acid in both the gender were statistically significant in no diabetic retinopathy than in NPDR. Our findings were in parallel to another study that showed serum uric acid level was much higher in male than female [41]. Evidence regarding estrogen promotes uric acid excretion [42] supports the findings of hyperuricemia among the males. Choi HK et al. [42], reported that serum uric acid was significantly higher in diabetic male than female. However, this sex predilection it’s still controversial. In contrary Causevic A et al. [35], demonstrated there is no effect of gender on serum uric acid levels in diabetic patient.

As we know, there are various studies that regard diabetic retinopathy as a neurovascular disease whereby the retinal neurodegeneration antedates the microvascular abnormalities [4, 43]. The retinal nerve fibre loss was contributed by retinal ganglion cell death and axonal degeneration. In our study, the mean RNFL thickness in all the 4 quadrants was thinner in no diabetic retinopathy compared to NPDR. Only the temporal quadrant was statistically significant. Our findings were parallel to some studies [26, 43] and in contrast to few other studies [44, 45]. All 9 subfields of the macular region showed similar changes as the RNFL. However, only 3 subfields were statistically significant (superior outer, inferior outer and temporal outer). These results of our studies were consistent with some studies [13, 46] and contradictory to few other studies [47, 48].

Several studies, including ours, showed presence of neuronal abnormalities at the early stage of diabetes irrespective of the type of optical coherence tomography (OCT) used [49, 50]. This explains the thinner retina in no diabetic retinopathy and the increase vascular permeability leads to increased retinal thickness. In our study, we have pooled all severity of NPDR grades, gender and age. Like findings found by other studies [25, 50], our study assumed that increase in the retinal thickening observed in the NPDR group was due to the insult from hard exudates and retinal hemorrhages leading to accumulation of intra-retinal fluids. This subsequently results in an increased thickness of the RNFL. Although we have excluded patients with clinically apparent and extensive retinal oedema, a possible presence of subclinical retinal oedema in our diabetic patients can interfere the OCT measurement. Subclinical macular oedema in which clinically shows absence of macular oedema on slit lamp examination but on OCT shows abnormally increased macular thickness [50].

We found that only the temporal RNFL quadrant showed a mean significant difference. The superior RNFL quadrant also showed a change in the retinal thickness between the no diabetic retinopathy and NPDR groups however it was not significant. The inferior RNFL quadrant showed a minimal change which is almost negligible between the two groups. In the macular thickness, only the outer most ring including the superior, temporal and inferior subfield showed a significant difference. Several studies have observed the RNFL of the superior and temporal quadrants to be thinner than that of the inferior quadrants [51, 52]. These results support the theory that early events of diabetic retinal disease [microaneurysms and acellular capillaries] occur preferentially in the superior temporal quadrant rather than the inferior quadrants. Chung et al. [53] demonstrated hypercapnia resulted in an increase in hemodynamic flow in the superior temporal quadrant. However, the response was not observed in a hyperoxic condition. In contrast, hyperoxia led to a decrease in blood flow to the inferior retina, whereas hypercapnia did not result in an increased flow within this area [53].

The lack of the normal vasoconstrictor response in the superior quadrant could explain; 1) the increased susceptibility of developing micro aneurysms and acellular capillaries in this region and 2) the preferential loss of retinal fibres in this region before diabetic retinopathy becomes clinically detectable [54]. Another study found the superior quadrant to be more predisposed to damage and a higher rate of cellular death, which resulted in RNFL thinning [26]. Our study demonstrated a lack of thinning of the RNFL at the nasal quadrants. This may be due to lack of micro aneurysmal development, hence sparing damage to the RNFL in this region. We assume the differences in the vascular hemodynamics in the macular region might be also the contributing factors to these phenomena of asymmetrical retinal thickness.

Serum uric acid pathogenesis, has been associated with the development and worsening of the diabetic retinopathy [31]. Chien KL et al. [55] and Ishizaka N et al. [56], suggested that increase level of serum uric acid acts as a predictor for diabetic vascular complications. Oxidative stress is a culprit for the progression of diabetic retinopathy [29]. Serum uric acid conversely can act as an antioxidant [31]. However, Jianfei Xia et al. [57] demonstrated that there was significantly increased concentration of uric acid among diabetic patients and serum uric acid might be a risk factor for diabetic retinopathy. High serum uric acid level was said as a risk factor of type 2 diabetes mellitus. Lowering the serum uric acid with xanthine oxidase inhibitors can reduce the incidence of type 2 diabetes mellitus and its complication but this is still controversial. Anju G et al. [58] in her studies concluded that serum uric acid levels increase in newly diagnosed diabetic patients, thus it can serve as potential biomarker of the glucose metabolism.

We did a correlation between the serum uric acid with RNFL thickness and macular thickness among no diabetic retinopathy and NPDR. Based on our Medline research, our study is the first study to be conducted. Uric acid inspite being an antioxidant in the circulation, it induces oxidative stress in the vascular endothelial cells, thus mediating progression of disease related to diabetic. Oxidative stress is believed to play an important role in the development of vascular complications in type 2 diabetes mellitus [21].

From our study, we did not find any momentous correlation between the RNFL and macular thickness with serum uric acid in both the groups. However, in those patients with no diabetic retinopathy, only the inferior outer macular subfield which had poor negative correlation with serum uric acid showed a statistically significant relationship. This negative relationship indicates that the higher serum uric acid level, the patient is likely to have minimal thinning of the macular region but the relationship is at a lower rate. This must be interpreted cautiously as only one macular subfield showed a significant relationship. From our findings, we postulate that, in no diabetic retinopathy group, the serum uric acid level is higher which is said to precede the type 2 diabetes, and inversely causes thinning of the macular region secondary to retinal neurodegeneration.

In those patients with NPDR, only the central subfield and inferior inner macula showed a fair positive correlation with a significant relationship. This positive significant relationship indicates that the higher serum uric acid level, the patient is likely to have thicker macular but the relationship is at a lower rate. From our findings, we postulate that, in NPDR group, there will be high likely for nephropathy to set in, thus affecting the renal excretion, increases rates of renal reabsorption, increases the excretion of purine metabolism subsequently raises the production of uric acid. The increase in serum uric acid level via the inflammatory process results in progression of diabetic vascular complication, thus causing vascular leakage and indirectly causing an impact to the macular thickness especially in the posterior pole.

There are several limitations found in our studies. The pooling of the severity of NPDR would have led to potential spurious comparisons. Other limitations include not considering the duration of diabetes, no follow up given, distribution of race and gender were not equal. We also did not include parameters for kidney dysfunction or diabetic nephropathy that could alter the excretion of uric acid.

## Conclusion

Serum uric acid showed a poor correlation with RNFL and macular thickness among type 2 diabetic patients. A large population cohort study with well distributed ethnicity and gender is needed to observe if there is presence of correlation between serum uric acid with RNFL and macular thickness among type 2 diabetic patients.

## Abbreviations

CI: Confidence interval
DR: Diabetic retinopathy
ERG: Electroretinogram
ETDRS: Early Treatment Diabetic Retinopathy Study
HbA1_C_: Glycosylated haemoglobin
NPDR: Non-proliferative diabetic retinopathy
OCT: Optical coherence tomography
RNFL: Retinal nerve fibre layer
SD: Standard deviation
SD-OCT: Spectral-Domain Optical Coherence Tomography
SPSS: Statistical Package for Social Sciences
